# A Rare Case of Non-Small-Cell Lung Carcinoma Complicated by Massive Pericardial Effusion and Cardiac Tamponade: A Case Report

**DOI:** 10.7759/cureus.44047

**Published:** 2023-08-24

**Authors:** Akbar Hussain, Opal Prevatt, Jonathan Piercy, Nazneen Ahmed, Stanley Marlowe, Georges Damaa

**Affiliations:** 1 Internal Medicine, Appalachian Regional Healthcare, Harlan, USA

**Keywords:** pericardiocentesis, metastasis, cardiac tamponade, pericardial effusion, small cell lung carcinoma

## Abstract

This case report presents a late middle-aged man with a right infra-hilar lung mass and pericardial effusion (PE). The patient was diagnosed with metastatic small-cell lung carcinoma, with metastases to the liver, pancreas, and cerebellum. The pericardial fluid cytology confirmed the presence of malignant cells most compatible with non-small-cell carcinoma. The patient received carbo/etoposide chemotherapy, and his treatment plan included adding atezolizumab and radiation therapy. Despite the excellent efficacy of immunotherapy, immune-related adverse events (IRAEs), including cardiac toxicity, were noted in some patients. PE related to immune checkpoint inhibitor (ICI) use is rare but potentially severe. This case highlights the importance of vigilant monitoring for cardiovascular symptoms during immunotherapy and the significance of pericardial fluid analysis in diagnosing malignant pericardial disease. Prompt diagnosis and appropriate treatment can lead to improved patient outcomes in cases of lung cancer-associated cardiac complications.

## Introduction

The most common cause of cancer-related death globally is lung cancer. Eighty-five percent of lung cancer cases are caused by non-small-cell lung cancer, and as most afflicted patients' cancers are well advanced when they are diagnosed, their prognosis is not good [1]. The parietal and visceral layers of the pericardium are typically separated by a modest amount of fluid (15-50 mL) [2]. Malignancy may result in pericardial effusion (PE), as tumor, surface, and lymphatic involvement that interferes with the body's natural reabsorption mechanism or increases fluid production may cause the effusion to develop [3].

Malignancy has been linked to serious cardiac complications such as cardiac tamponade and PE. Exertional dyspnea and a progressive inability to exercise may be symptoms of chronic PE. If a chronic PE becomes severe, it may result in cardiac tamponade and an immediate hemodynamic compromise that calls for immediate treatment [4]. A potentially fatal cardiac tamponade may be brought on by a condition known as carcinomatous pericarditis. Increased intrapericardial pressure, which results from the hemodynamic consequences of pericardial fluid buildup, impairs diastolic filling and reduces cardiac stroke volume. Shock and cardiac arrest can happen suddenly in specific situations. Prompt and appropriate treatment of PE and/or cardiac tamponade can reduce the rate of these potentially fatal complications [5].

Among various treatment modalities, immune checkpoint inhibitor (ICI)-based immunotherapies have been proven effective in treating various cancers, with anti-PD-1/PD-L1 complementing conventional chemotherapies. However, immune-related adverse events (IRAEs), including cardiac toxicity, are becoming more common. Fewer than 1% of patients experience significant hemodynamic impairment due to PE. It's interesting to note that none of these individuals had a myocardial illness, which prompted some writers to use the more precise term pericardial-only ICI-associated disease [6,7].

We present a case of a 62-year-old male with extensive-stage non-small-cell lung carcinoma who presented with symptoms of chest pain and shortness of breath and was diagnosed with PE and metastases. The treatment plan involves carbo/etoposide chemotherapy, atezolizumab immunotherapy, and radiation therapy, with close monitoring for response and side effects.

## Case presentation

**Figure 1:**
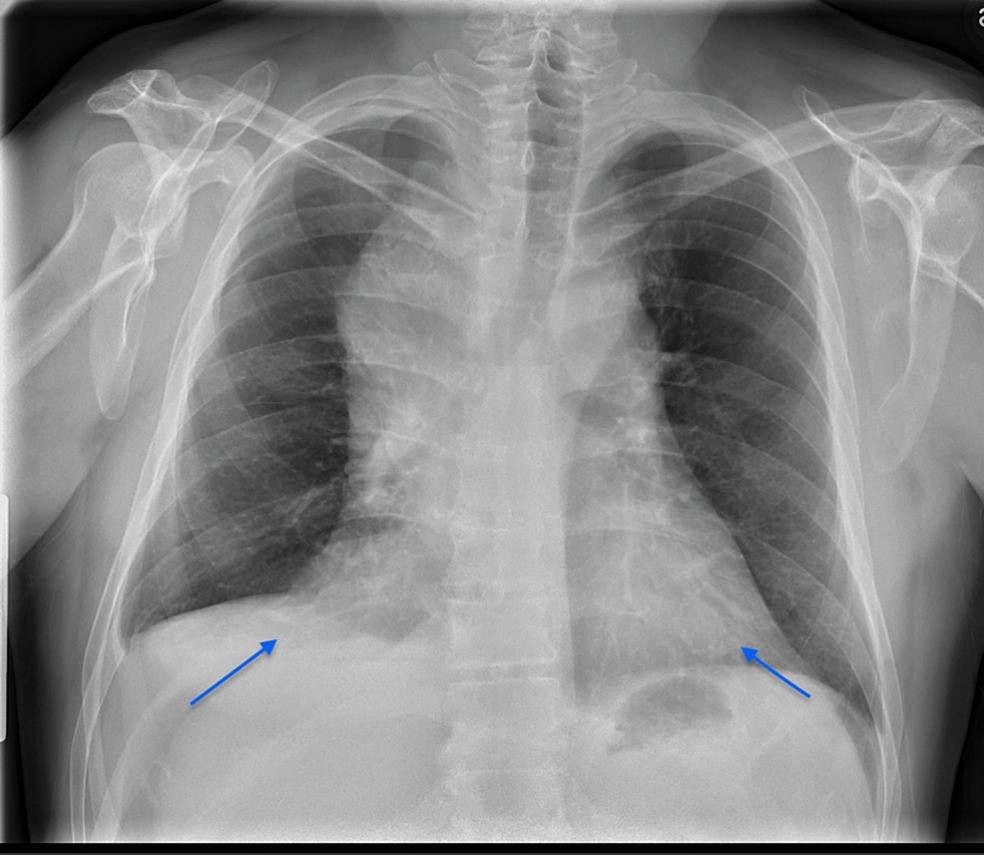
Chest X-ray showing bilaterally enlarged cardiac silhouette (arrows)

We present the case of a 62-year-old male who was referred from a previous facility for further treatment of extensive-stage non-small-cell lung carcinoma. The patient's symptoms had been present for six months and was admitted to our hospital with right-sided chest pain, shortness of breath, and orthopnea. Upon examination, the patient displayed a healthy appearance and showed no immediate signs of distress. He was on continuous oxygen therapy at a rate of 2 liters per minute and had diminished lung sounds on the right. The remainder of the physical examinations were unremarkable, with no significant findings in the eyes, neck, cardiovascular system, gastrointestinal system, or skin. Chest X-ray revealed a bilateral enlarged cardiac silhouette which raised suspicion for PE as shown in Figure 1.

**Figure 2:**
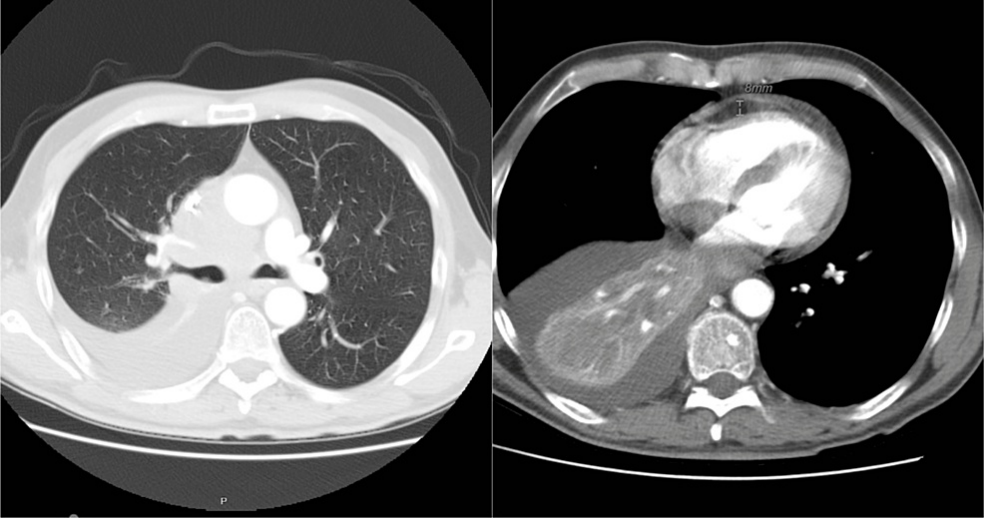
A CT of the chest showed pleural effusion (A) and PE on the right side, as well as a right infra-hilar mass, mediastinal adenopathy, and hilar adenopathy (B)

**Figure 3:**
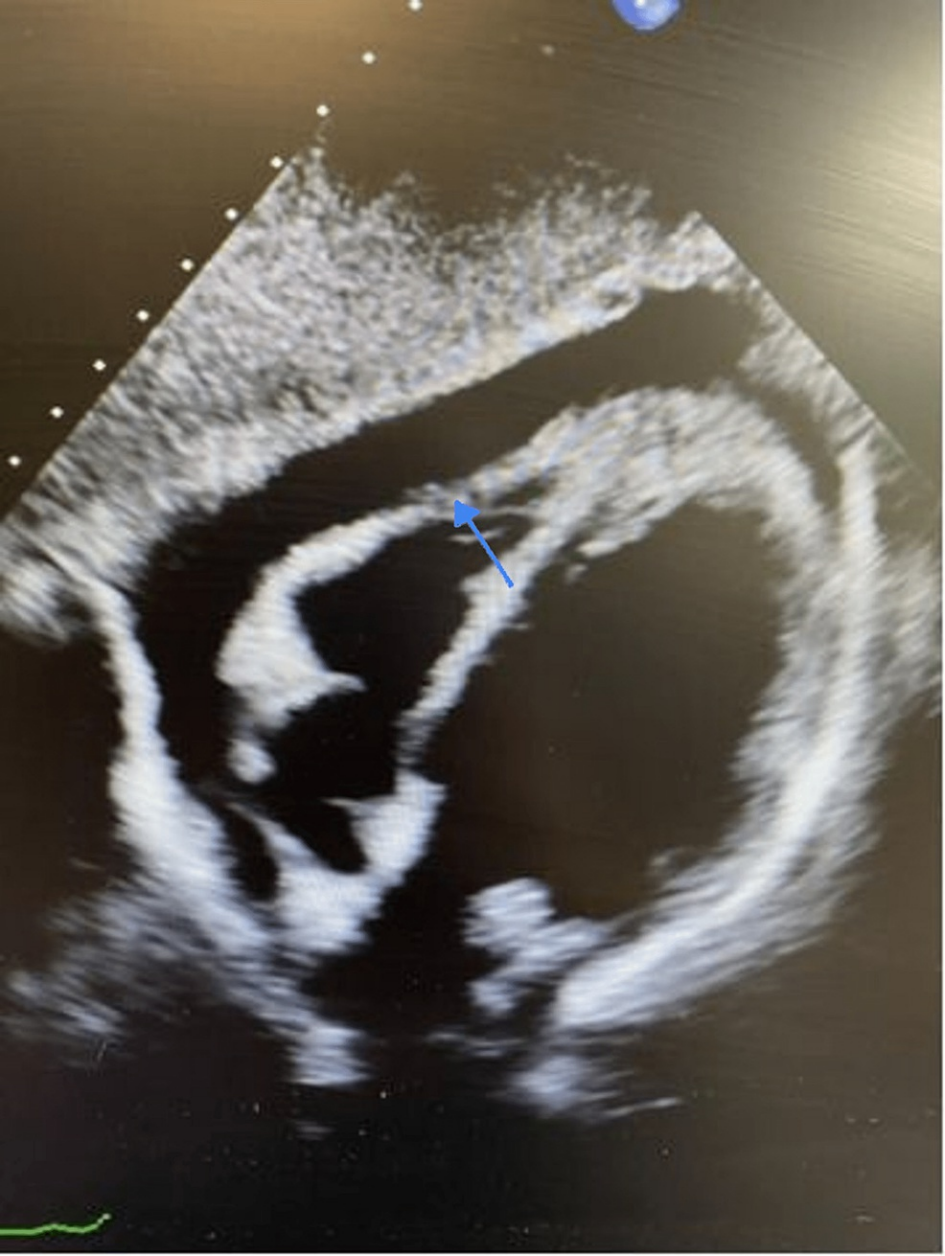
Severe PE on apical four-chamber echocardiography

A CT chest revealed a right infra-hilar mass, mediastinal and hilar adenopathy, as well as a right-sided pleural effusion and PE as shown in Figure 2. On follow-up, subsequent echocardiography showed massive PE with tamponade physiology dimensions of anterior 1.2 cm, lateral 3.0 cm, and posterior 2.5 cm, along with normal valve and chamber size as shown in Figure 3. Further diagnostic procedures, including thoracentesis and pericardiocentesis, were performed, and the pericardial fluid cytology confirmed the presence of malignant cells most compatible with non-small-cell carcinoma.

Subsequent imaging studies, including an MRI of the abdomen and head, showed evidence of metastatic disease in the liver, pancreas, and cerebellum. The patient received one cycle of carbo/etoposide chemotherapy during his hospital stay. At the follow-up visit, he reported mild improvement in shortness of breath and cough, although he continued to experience pain in the right lower chest, which remained uncontrolled with over-the-counter medications.

After a comprehensive discussion, the patient was educated about the aggressive nature of metastatic non-small-cell carcinoma affecting the liver, pancreas, and cerebellum, along with the potential advantages and drawbacks of chemotherapy and radiation therapy. Subsequently, the treatment strategy was devised to encompass the continuation of carbo/etoposide chemotherapy, the introduction of atezolizumab immunotherapy, and a referral for radiation oncology assessment.

The patient agreed to the proposed treatment plan and demonstrated an understanding of the risks and benefits involved. Their follow-up appointment was scheduled in four weeks to assess the response to treatment and evaluate the need for further interventions. On follow-up, the patient responded well to the treatment given.

## Discussion

A combination of autopsy series reveals that 15.4% of cancer autopsies find cardiac metastatic disease, suggesting that the heart might be involved in neoplastic illness [8]. A cardiac tamponade affects 16% of these individuals with cardiac metastases [9]. When primary lung cancer spreads to the heart, the pericardium is where it does so most frequently. The fundamental basis for the investigation of PE involves cytologic assessment, particularly in instances of hemorrhagic specimens lacking a traumatic etiology, as such cases are more inclined to indicate malignancy. The analytical sensitivity for malignancy identification ranges from 67% to 92%, with some investigations reporting high specificities of over 100%. When paired with optical microscopy, the integration of useful fluorescence data from hematology analyzers, such as the XN series (Sysmex), UniCel DxH (Beckman Coulter, CA), or Sysmex, might be successful for malignancy diagnosis. A pericardial biopsy may be necessary for the definitive diagnosis when no cancer cells are discovered in the fluid or when a pericardiocentesis cannot be performed because of low-volume effusions. If the pericardial fluid does not contain sufficient cells to do a cell block, a pericardial biopsy may be necessary for diagnostic immunocytology or molecular testing [10].

Clinically speaking, immunotherapy's cardiovascular toxicities, albeit uncommon, may be severe and have a surprisingly high risk of fatal consequences [11-13]. The true prevalence of this toxin is yet unknown; thus, it's important to account for any potential underestimation. This may be due to several factors, including the following: (a) the absence of data on this specific side effect from pivotal clinical trials; (b) a still limited clinical experience; (c) clinicians' lack of attention to cardiovascular symptoms that may be mistaken for disease progression, particularly in patients with lung cancer, who represent the majority of cancer patients receiving ICIs; and (d) a failure to recognize the clinical presentation of cardiac IRAEs. Even the extensive pharmacovigilance analysis that was just published may have been skewed by practitioners' lack of interest in and resistance to reporting adverse effects [14].

Malignant pericardial disease is identified by irregular pericardial thickness and mediastinal lymphadenopathy [15]. Pericardial fluid analysis is crucial to distinguish between the two diagnoses since individuals with tuberculosis of the pericardium might also exhibit irregular pericardial thickness. Since most pericardial metastases arise from the mediastinal lymph nodes, mediastinal lymphadenopathy is present [16]. Patients with moderate to significant PE and clinical suspicion of cancer should undergo pericardiocentesis [17]. Pericardiocentesis is commonly used to treat symptomatic PE, serving both therapeutic and diagnostic purposes. With high recurrence rates, pericardiocentesis alone is insufficient for long-term palliation. Surgery is often only performed when the buildup is fast or the pericardiocentesis is insufficient [18]. Sclerosing agents, for example, were adjuncts to pericardiocentesis that did not provide any benefits but rather had fatal adverse effects [3]. On the best course of action for treating cardiac tamponade, there is no universal agreement. The currently used techniques include pericardiopleural window formation, pericardial catheter insertion, pericardiocentesis, and external radiation treatment. It has been previously detailed how these techniques work in conjunction with intraperitoneal instillation of chemotherapeutic or sclerosing drugs [19,20].

Compared to pericardial biopsy, pericardial fluid cytology has a higher diagnostic value for identifying cancer [21]. With varied sensitivity, pericardial fluid cytology has 100% specificity. In more than half of malignant patients, pericardial fluid cytology revealed nonmalignant cells, most likely brought on by opportunistic infections, radiation, or chemotherapy [3]. Advanced malignancy is typically indicated by malignant PE. Patients with positive malignant pericardial fluid cytology had a median survival time of 7 to 15 weeks. The median survival rate for non-malignant pericardial fluid cytology is substantially higher [3].

## Conclusions

This case report addresses the management challenges of advanced non-small-cell lung carcinoma with pericardial involvement. The patient's severe symptoms, diagnostic findings, and treatment plan underscore the importance of recognizing cardiac complications in advanced lung cancer. The effectiveness of ICI requires vigilance in managing potential cardiac toxicities. A multidisciplinary approach, including chemotherapy, immunotherapy, and close monitoring, is crucial for comprehensive care and improved patient outcomes.

## References

[1] Siegel RL, Miller KD, Jemal A (2016). Cancer statistics, 2016. CA Cancer J Clin.

[2] Kasper DL, Fauci AS, Hauser SL, Longo DL, Jameson J, Loscalzo J (2016). Pericardial disease. https://accessmedicine.mhmedical.com/content.aspx?bookid=1820&sectionid=127557882.

[3] Schusler R, Meyerson SL (2018). Pericardial disease associated with malignancy. Curr Cardiol Rep.

[4] Ghosh AK, Crake T, Manisty C, Westwood M (2018). Pericardial disease in cancer patients. Curr Treat Options Cardiovasc Med.

[5] Jensen JK, Poulsen SH, Mølgaard H (2017). Cardiac tamponade: a clinical challenge. ESC.

[6] Canale ML, Camerini A, Casolo G (2020). Incidence of pericardial effusion in patients with advanced non-small cell lung cancer receiving immunotherapy. Adv Ther.

[7] Palaskas N, Morgan J, Daigle T (2019). Targeted cancer therapies with pericardial effusions requiring pericardiocentesis focusing on immune checkpoint inhibitors. Am J Cardiol.

[8] Mukai K, Shinkai T, Tominaga K, Shimosato Y (1988). The incidence of secondary tumors of the heart and pericardium: a 10-year study. Jpn J Clin Oncol.

[9] Onuigbo WI (1974). The spread of lung cancer to the heart, pericardium and great vessels. Jpn Heart J.

[10] Saab J, Hoda RS, Narula N (2017). Diagnostic yield of cytopathology in evaluating pericardial effusions: clinicopathologic analysis of 419 specimens. Cancer Cytopathol.

[11] Varricchi G, Galdiero MR, Marone G (2017). Cardiotoxicity of immune checkpoint inhibitors. ESMO Open.

[12] Johnson DB, Balko JM, Compton ML (2016). Fulminant myocarditis with combination immune checkpoint blockade. N Engl J Med.

[13] Altan M, Toki MI, Gettinger SN (2019). Immune checkpoint inhibitor-associated pericarditis. J Thorac Oncol.

[14] Salem JE, Manouchehri A, Moey M (2018). Cardiovascular toxicities associated with immune checkpoint inhibitors: an observational, retrospective, pharmacovigilance study. Lancet Oncol.

[15] Sun JS, Park KJ, Kang DK (2010). CT findings in patients with pericardial effusion: differentiation of malignant and benign disease. AJR Am J Roentgenol.

[16] Szturmowicz M, Pawlak-Cieślik A, Fijałkowska A (2017). The value of the new scoring system for predicting neoplastic pericarditis in the patients with large pericardial effusion. Support Care Cancer.

[17] Adler Y, Charron P, Imazio M (2015). 2015 ESC Guidelines for the diagnosis and management of pericardial diseases: the Task Force for the Diagnosis and Management of Pericardial Diseases of the European Society of Cardiology (ESC)Endorsed by: The European Association for Cardio-Thoracic Surgery (EACTS). Eur Heart J.

[18] Gornik HL, Gerhard-Herman M, Beckman JA (2005). Abnormal cytology predicts poor prognosis in cancer patients with pericardial effusion. J Clin Oncol.

[19] Gregory JR, McMurtrey MJ, Mountain CF (1985). A surgical approach to the treatment of pericardial effusion in cancer patients. Am J Clin Oncol.

[20] Flannery EP, Gregoatos G, Corder MP (1975). Pericardial effusions in patients with malignant diseases. Arch Infern Med.

[21] Dragoescu EA, Liu L (2013). Pericardial fluid cytology: an analysis of 128 specimens over a 6-year period. Cancer Cytopathol.

